# Extended T^2 ^tests for longitudinal family data in whole genome sequencing studies

**DOI:** 10.1186/1753-6561-8-S1-S40

**Published:** 2014-06-17

**Authors:** Yiwei Liu, Jing Xuan, Zheyang Wu

**Affiliations:** 1 Department of Mathematical Sciences, Worcester Polytechnic Institute, 100 Institute Road, Worcester, MA 01609-2280, USA

## Abstract

Family data and rare variants are two key features of whole genome sequencing analysis for hunting the missing heritability of common human diseases. Recently, Zhu and Xiong proposed the generalized T^2 ^tests that combine rare variant analysis and family data analysis. In similar fashion, we developed the extended T^2 ^tests for longitudinal whole genome sequencing data for family-based association studies. The new methods simultaneously incorporate three correlation sources: from linkage disequilibrium, from pedigree structure, and from the repeated measures of covariates. We assess and compare these methods using the simulated data from Genetic Analysis Workshop 18. We show that, in general, the extended T^2 ^tests incorporating longitudinal repeated measures have higher power than the single-time-point T^2 ^tests in detecting hypertension-associated genome segments.

## Background

Compared with traditional genome-wide association studies (GWAS), whole genome sequencing (WGS) is a more efficient way of finding the missing heritability of diseases [1]. While GWAS are mostly based on microarray genotyping, which can discover only common genetic variants, WGS is able to reveal rare and structural variants, which are crucial factors behind disease phenotypes [2]. As the cost of sequencing decreases significantly, we expect that WGS will become increasingly predominant in the hunt for novel disease genes.

Most of the recent discoveries from sequencing studies were based on the Mendelian trait model [3]. Genetic association studies based on the complex trait model are challenging because of limited sample size as well as the new properties of sequencing data. WGS data are distinct from GWAS data in two major aspects. First, WGS provides a huge number of rare variants. Even with large allelic effects, caused by very small minor allele frequencies (MAFs), the association tests between single rare variants and the trait are less powerful and unreliable [4]. Second, family designs play a critical role in WGS. Because of its relatively high cost, WGS tends to exploit families of patients, so that the rare causal variants are likely enriched through cotransmission of the disease [5]. Furthermore, the pedigree structure allows statistical imputation of the genotypes at no experimental cost, which potentially increases the statistical power [6,7].

In a recent celebrated work, Zhu and Xiong proposed a set of generalized T^2 ^tests for family-based WGS data [8]. These methods simultaneously address the correlations among genetic variants (i.e., linkage disequilibrium [LD]) and the correlations among family members (i.e., kinship). Rare-variant collapsing procedures [9,10] are also integrated into the tests. However, these methods cannot incorporate covariates and do not address the correlation structure for longitudinal repeated measures. In this study, we further extended the methodology of the T^2 ^tests to address these limits. By applying these methods to an analysis of the Genetic Analysis Workshop 18 (GAW18) simulation data, we showed that the asymptotic null distributions of Zhu and Xiong [8] are problematic in controlling the type I error rate, and that our extended methods are likely more powerful for longitudinal data.

## Methods

### Generalized T^2 ^tests for family data

Zhu and Xiong [8] showed that the covariance as a result of both LD and kinship could be explicitly expressed as a Kronecker product of the corresponding covariance matrices. Following the idea of Hotelling's T^2 ^test [11,12], the authors proposed a generalized T^2 ^test that incorporates these covariance matrices, which are estimated separately by using the same data. Depending on various strategies of collapsing of rare variants, here we consider three generalized T^2 ^tests of Zhu and Xiong.

**T^2^**: The genotypes of rare variants between adjacent common variants are summed up, and one covariance matrix is estimated for both common and collapsed rare variants.

**CMC.ZXpaper (CMC test)**: The rare variants are collapsed in the same way as above, but the covariance matrices are estimated separately for common and rare variants (assuming they are uncorrelated).

**CMC.ZXcode**: Rare variants are collapsed by the maximum of their genotypes, and one covariance matrix is estimated for both common and collapsed rare variants. This strategy follows the R function pedCMC of Zhu and Xiong (https://sph.uth.edu/hgc/faculty/xiong/software-D.html).

### Extended T^2 ^test for family data with longitudinal repeated measures

**Figure 1:**
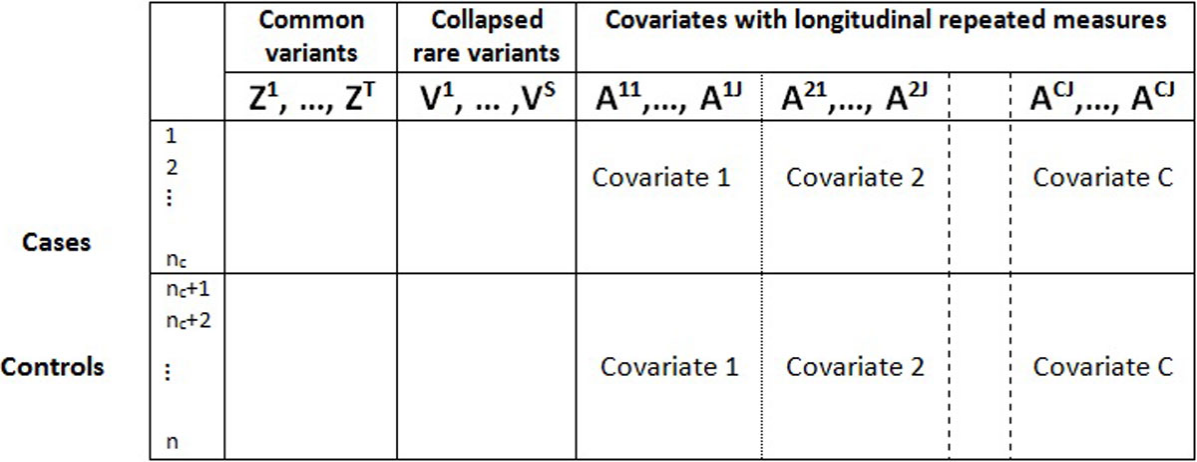
**Data structure for composing the extended T^2 ^tests**. Data contain 3 blocks: common variants, rare variants, and longitudinal covariate measures. The statistics integrate the correlations among both rows and columns, and test whether there exists a significant difference between the row vector mean of the cases and that of the controls.

Building on the idea of Zhu and Xiong, we further extend the generalized T^2 ^tests to account for the longitudinal repeated covariates. Figure 1 shows the data structure and the idea of the extension. Specifically, the extended T^2 ^tests compare the blocks of common variants, rare variants, and covariates with repeated measures in cases and in controls, while simultaneously accounting for the correlations among genetic factors, among pedigree individuals, and among longitudinal repeated measures. The response is the occurrence of the event at any of the measurement points.

Following the notations in Figure 1, let $n_{c}$ be the number of the cases, $n_{d}$ be the number of the controls, and $n=n_{c}+n_{d}$. The genotype column vector of the *t*th common variant is $Z^{t}={\left(Z_{1}^{t},\ldots ,Z_{n}^{t}\right)}^{\prime }$, the aligned column vector of all *T *common variants is represented by $Z={\left({Z^{1}}^{\prime },\ldots ,{Z^{T}}^{\prime }\right)}^{\prime }$. Similarly, for the collapsed genotypes of rare variants, the genotype column vector of the *s*th rare variant is $V^{s}={\left(V_{1}^{s},\ldots ,V_{n}^{s}\right)}^{\prime }$, and $V={\left({V^{1}\prime }^{},\ldots ,{V^{S}\prime }^{}\right)}^{\prime }$ for totally *S *rare variants. Considering the covariates with longitudinal repeated measures, the column vector of the *c*th covariate at the *j*th repeated measurement point is $A^{cj}={\left(A_{1}^{cj},\ldots ,A_{n}^{cj}\right)}^{\prime }$, and the aligned column vector is $A={\left({A^{11}\prime }^{},\ldots ,{A^{1J}\prime }^{},{A^{21}\prime }^{},\ldots ,{A^{2J}\prime }^{},\ldots ,{A^{C1}\prime }^{},\ldots ,{A^{CJ}\prime }^{}\right)}^{\prime }$ for totally *C *covariates, each measured for *J *times. Similarly, the row vectors are denoted as follows. For i=1,…,n, the vectors $Z_{i}={\left(Z_{i}^{1},\ldots ,Z_{i}^{T}\right)}^{\prime }$ are the rows in the block of common variants, the row vectors $V_{i}={\left(V_{i}^{1},\ldots ,V_{i}^{S}\right)}^{\prime }$ are for rare variants, and $A_{i}={\left(A_{i}^{11},\ldots ,A_{i}^{1J},A_{i}^{21},\ldots ,A_{i}^{2J},\ldots ,A_{i}^{C1},\ldots ,A_{i}^{CJ}\right)}^{\prime }$ are for longitudinal covariates. The row average in cases is ${\bar{Z}}_{c}=\sum_{i=1}^{n_{c}}Z_{i}/n_{c}$, and that in controls is ${\bar{Z}}_{d}=\sum_{i=n_{c}+1}^{n}Z_{i}/n_{d}$. The row averages for rare variants and covariates are obtained analogously.

The idea of the extended T^2 ^test is simply to compare the difference between the row average of the case blocks and the row average of the control blocks. Let $\eta ={\left(Z^{\prime },V^{\prime },A^{\prime }\right)}^{\prime }$. The difference between row averages can be written in terms of *η*. That is

(1) $$H\eta =\frac{n_{c}n_{d}}{n}\left(\begin{array}{c} {\bar{Z}}_{c}-{\bar{Z}}_{d}\\ {\bar{V}}_{c}-{\bar{V}}_{d}\\ {Ā}_{c}-{Ā}_{d} \end{array}\right),$$

where if we define $D_{r}={\left(u_{1},\ldots ,u_{n}\right)}^{\prime }$, with $u_{i}=1$ for cases $i=1,\ldots ,n_{c}$ and $u_{i}=0$ for controls $i=n_{c}+1,\ldots ,n$, and denote 1 as a vector of 1 of length *n *and $I_{\left(k\right)}$ as an identity matrix of dimension *k*, then the matrix

(2) $$H=\left(\begin{array}{ccc} I_{\left(T\right)}⊗\left(D_{r}-\frac{n_{c}}{n}1\right) & 0 & 0\\ 0 & I_{\left(S\right)}⊗\left(D_{r}-\frac{n_{c}}{n}1\right) & 0\\ 0 & 0 & I_{\left(CJ\right)}⊗\left(D_{r}-\frac{n_{c}}{n}1\right) \end{array}\right).$$

Following the idea of the generalized T^2 ^test, the extended T^2 ^test is $T^{2}={\left(H\eta \right)}^{\prime }{\text{Γ}}^{-1}\left(H\eta \right),$ where $\text{Γ}=\text{Var}\left(H\eta \right)=H\text{Λ}H^{\prime }$, $\text{Λ}=Var\left(\eta \right).$

The key problem is to estimate Λ. Following the assumption of Zhu and Xiong [8] that *Z *and *V *are independent, we consider two cases. In the first case, assume *A *is also independent with *Z *and *V*. Then $Var\left(\eta \right)=\text{Λ}=diag\left({\text{Λ}}_{Z},{\text{Λ}}_{V},{\text{Λ}}_{A}\right)$, whereby

${\text{Λ}}_{Z}=Var\left(Z\right)={\text{Σ}}_{Z}⊗\text{Φ}$ and ${\text{Λ}}_{V}=Var\left(V\right)={\text{Σ}}_{V}⊗\text{Φ}$.

${\text{Σ}}_{Z}$ and ${\text{Σ}}_{V}$ are the covariance matrix among the elements in $Z_{i}$ and $V_{i}$, respectively (e.g., the LD among the genetic variants), *Φ *is the kinship matrix, and ⊗ denotes the Kronecker product. For the covariate block, we consider ${\text{Λ}}_{A}=Var\left(A\right)={\text{Σ}}_{A}⊗{\text{Φ}}^{*}$, where ${\text{Σ}}_{A}$ is the covariance matrix among the elements $A_{i}$, and ${\text{Φ}}^{*}$ is a matrix that captures the correlations among individuals in terms of environmental covariates.

To better account for the heterogeneity of the data in cases and in controls, we applied the method in Hotelling's T^2 ^test for estimating the covariance matrix (which is different from equation (6) in Ref. [8]). Then equation (3) is simplified to

(3) $$T^{2}={\left(\frac{n_{c}n_{d}}{n}\right)}^{2}\left[\frac{{\left({\bar{Z}}_{c}-{\bar{Z}}_{d}\right)}^{\prime }{\hat{\text{Σ}}}_{Z}^{-1}\left({\bar{Z}}_{c}-{\bar{Z}}_{d}\right)+{\left({\bar{V}}_{c}-{\bar{V}}_{d}\right)}^{\prime }{\hat{\text{Σ}}}_{V}^{-1}\left({\bar{V}}_{c}-{\bar{V}}_{d}\right)}{{\left(D_{r}-\frac{n_{c}}{n}1\right)}^{\prime }\text{Φ}\left(D_{r}-\frac{n_{c}}{n}1\right)}+\frac{{\left({Ā}_{c}-{Ā}_{d}\right)}^{\prime }{\hat{\text{Σ}}}_{A}^{-1}\left({Ā}_{c}-{Ā}_{d}\right)}{{\left(D_{r}-\frac{n_{c}}{n}1\right)}^{\prime }{\text{Φ}}^{*}\left(D_{r}-\frac{n_{c}}{n}1\right)}\right].$$

We consider two simplification assumptions: (a) ${\text{Φ}}^{*}=I$ indicates that covariate variables among individuals are independent, considering the individual dependence has been captured by the genetics; and (b) ${\text{Φ}}^{*}=\text{Φ}$ indicates that covariate variables among individuals have the similar dependence pattern as that according to genetics (e.g., children may be more likely to smoke if parents do, or the age of children is correlated with the age of parents). According to the various rare-variant collapsing strategies in the above generalized T^2 ^tests by Zhu and Xiong [8], the corresponding extended T^2 ^tests are denoted T2.longi, CMC.ZXpaper.longi, and CMC.ZXcode.longi, respectively.

### Asymptotic and permutation tests

Zhu and Xiong derived asymptotic chi-square distribution for the null. In their paper [8], the degrees of freedom (DF) equal the number of variants; in their R code, the DF equal the rank of data matrix. The latter is better but still gives inflated *p *values as shown below. Thus, we applied a permutation test for the type I error rate being well controlled. Specifically, let $T_{g}^{2}$ and $T_{gl}^{2}$, g=1,…,G, l=1,…,L, denote the test statistics of the *g*th genome window from the original data and from the *l*th permutation, respectively. The empirical *p *value for the *g*th window is $p_{g}=\#\left\{T_{gl}^{2}\geq T_{g}^{2},l=1,\ldots ,L\right\}/L$, where *L *=1000. Because the target is to find the associations with genetic variants, not with the covariates, the permutations are applied only to the genotype data to retain the relationship between response and the covariates.

## Results

For evaluating the above methods, we used the "dose" genotype data of 1,215,399 single-nucleotide variants (SNVs) on chromosome 3 and the 200 simulation replicates of hypertension outcomes and covariates (age, hypertension medicine use, smoking status). As an arbitrary, yet simple, way to group variants, we split chr3 into 19,080 windows, each 10 kilobase pairs (kbp) long. In each window, rare variants (MAF <0.05) between adjacent common variants were collapsed, leaving 654,415 genetic factors (common or rare variants, or collapsed rare-variant groups) to be analyzed. The average number of genetic factors contained in the windows is 34.3, the median is 32, the minimum is 1, and the maximum is 330. For the simulated phenotypes, the number of individuals is 849 in 20 families, where the family sizes are from 21 to 74, with the mean 42.45 and the median 36.5. There are 188 simulated true SNVs contained in 129 true windows (1, 3, 7, 32, and 86 windows contain 5, 4, 3, 2, and 1 true SNVs, respectively) on chr3. The knowledge of these true SNVs was used only for evaluating the power of these association tests, not for designing data analysis strategy.

**Figure 2:**
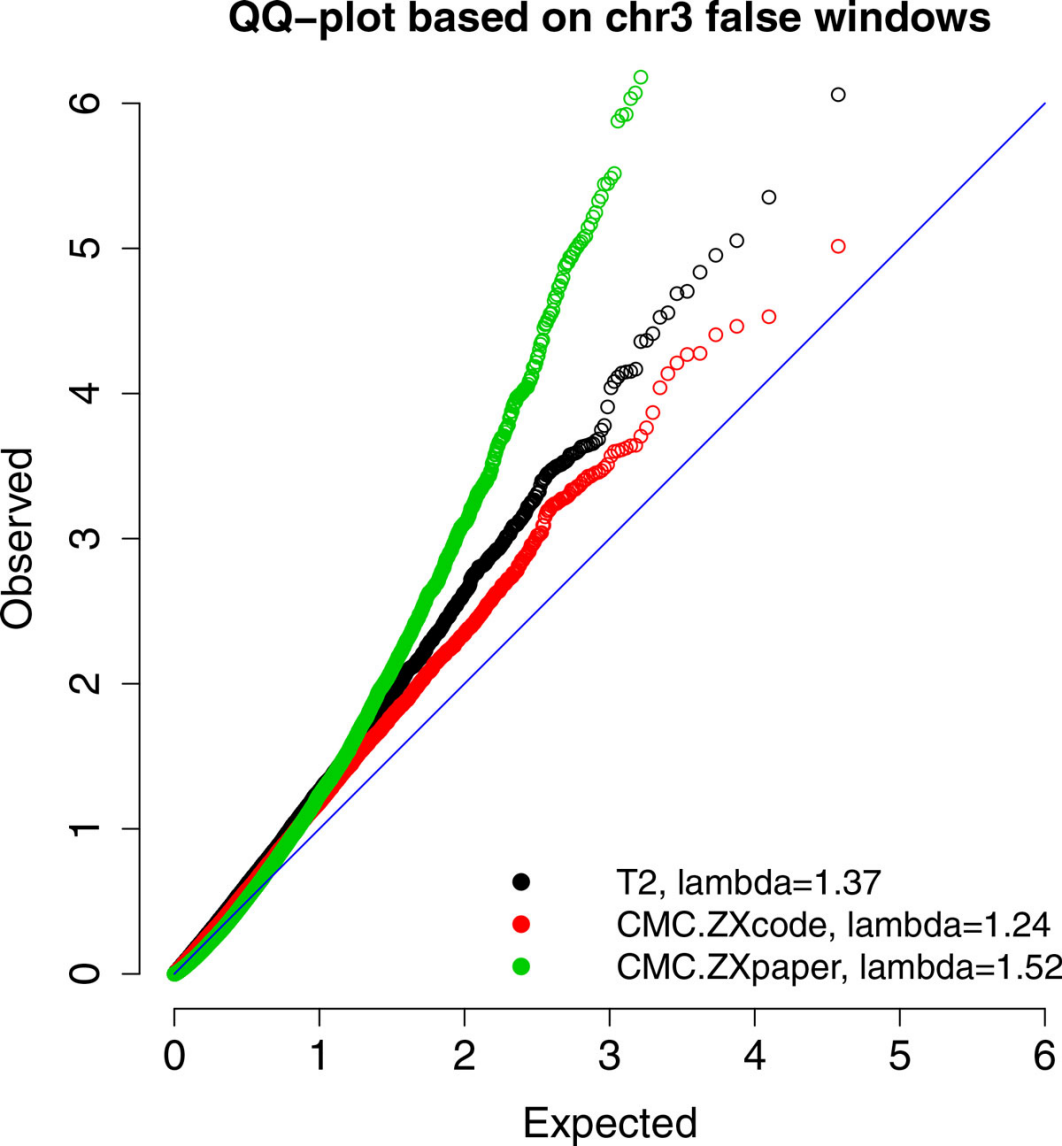
**Q-Q plot for asymptotic *p *values of Zhu and Xiong's generalized T^2 ^tests**. Results are based on the 18,951 false 10 kbp-long windows on chr3.

To assess the asymptotic null distributions of the tests provided in Zhu and Xiong [8], we obtained the asymptotic *p *values of these tests for all false windows in chr3. The Q-Q plot of Figure 2 shows that all three methods have inflated *p *values with large genomic inflation factors *λ*[13]. For example, when one chooses a *p *value cutoff of 0.05, the actual (empirical) error rate is approximately 0.1. At the same time, the following results show that permutation test controls the type I error rate well. Thus, the inflated type I error rate is likely caused by the inappropriate asymptotic null distributions, not by possible stratification.

**Figure 3:**
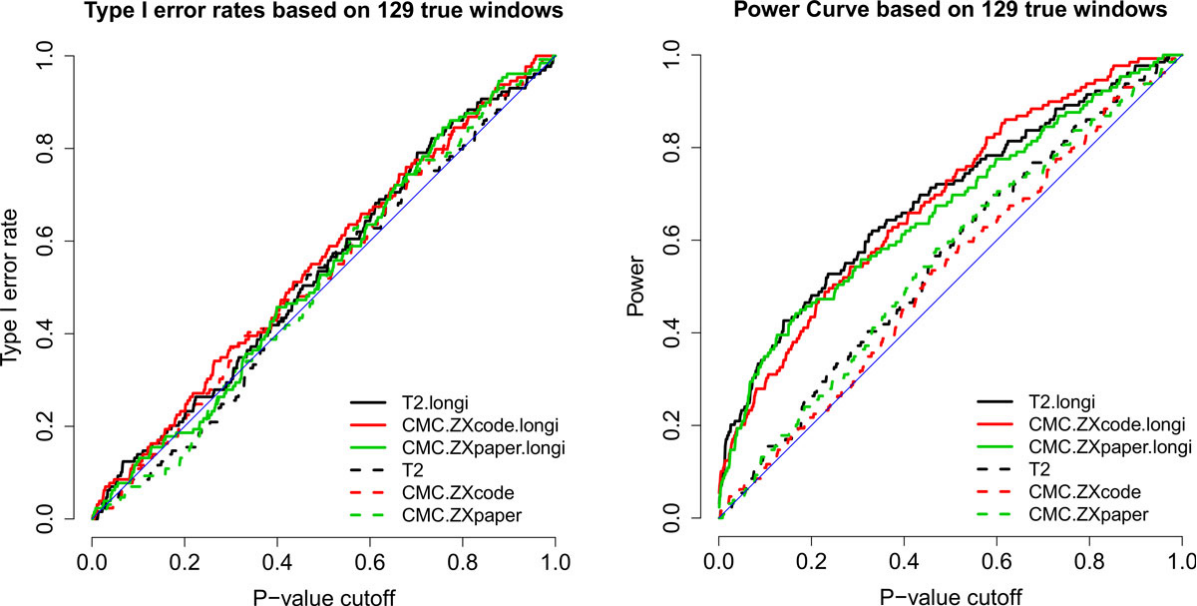
**Type I error rate and power of detecting true windows on chr3**. The power is the percentage of all 129 true windows that are detected at various *p *value cutoffs. The type I error rate is the same percentage of these windows, except the genotypes were permuted to destroy the genetic associations.

We studied the power of these tests in detecting true windows over chr3. Based on the phenotype data in the simulation replicate 1, the right panel of Figure 3 shows the receiver operating characteristic (ROC) curve for power (estimated by the true positive rate) over a variety of *p *value cutoffs. In general, the power is low at small or moderate *p *values. This phenomenon indicates that the sample size is still relatively too small for detecting many weak genetic effects simulated in the data. At the same time, it is clear that the 3 extended T^2 ^tests that incorporate longitudinal information are significantly better than the generalized T^2 ^tests that only consider the measures at the first time point. Because the two setups: ${\text{Φ}}^{*}=I$ and ${\text{Φ}}^{*}=\text{Φ}$ in (3) led to similar results, we only report that by ${\text{Φ}}^{*}=I$ for simplicity. The left panel of Figure 3 shows that the permutation test controls the type I error rates well for all methods.

**Figure 4:**
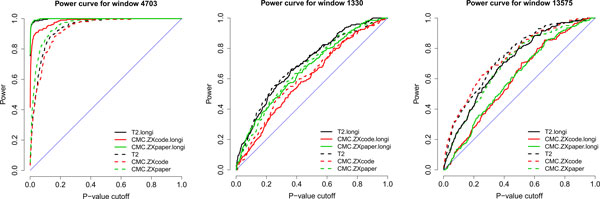
**Three patterns of comparisons between the longitudinal tests and one-time-point tests**. *Left: *longitudinal tests are better (illustrated by window 4703, 79 windows in total); *middle: *they are similar (illustrated by window 1330, 19 windows in total); *right: *one-time-point tests are better (illustrated by window 13575, 5 windows in total).

To compare the overall capabilities of these tests, we studied their power (i.e., true positive rates) in detecting each of all windows over 200 simulation replicates. As illustrated in Figure 4, there are 4 representative patterns of the comparisons for the 129 true windows on chr3. In particular, 93 windows have longitudinal extended T^2 ^tests more powerful than generalized T^2 ^tests (illustrated in Figure 4, left panel), 5 windows have similar results for both (Figure 4, middle panel), 15 windows have generalized T^2 ^tests more powerful (Figure 4, right panel), and the remaining 16 windows have almost no power for any tests. So, the longitudinal extended T^2 ^tests are significantly superior to the single-time-point generalized T^2 ^tests (93 vs. 15, *p *value = 3.8e-15 based on binomial model). For all windows, the type I error rates of all methods were well controlled (results are available upon request).

## Discussion

In the simulation data of GAW18, true SNVs are always allocated on genes. Using genes as windows to group SNVs may concentrate the true SNVs and has the potential to improve the detection power. However, the idea of WGS, instead of exome sequencing, is that the disease-related genetic factors might allocate outside of genes. So we did not use the knowledge that true SNVs are in genes; instead, we evaluated the methods based on fixed-genome segment windows.

There are several limitations and future research topics based on the current work. First, matrix estimation is a key issue in this methodology development. Good estimation of matrices and their inverses can better incorporate correlation structures' potential to improve the performance. Here we simply adopted the same variance matrix estimate in Hotelling's T^2 ^test. This is a maximal likelihood estimate if observations are independent. Unfortunately, independency is not true for family data in the first place. Besides the correlation issue, for a high-dimensional matrix with a potentially sparse structure, there are better estimates of the covariance matrix and its inverse [14]. Second, the permutation test is relatively slow, especially for handling large amounts of data in WGS. It would be desirable to derive more accurate asymptotic distributions for fast and precise *p *value calculation. Third, necessary modifications of these tests are needed to handle missing data and unequal numbers of repeated measures, which are common problems.

## Conclusions

We have extended Zhu and Xiong's [8] generalized T^2 ^tests to incorporate the covariates with longitudinal repeated measures. These methods account for 3 sources of correlation structures among genetic variants, family members, and time series observations. Compared with the T^2 ^test methods for snapshot observations, the new methods have higher power to detect hypertension-related genome segments according to the GAW18 simulation data.

## Competing interests

The authors declare that they have no competing interests.

## Authors' contributions

ZW designed the overall study. ZW, YL, and JX conducted statistical analyses and drafted the manuscript. All authors read and approved the final manuscript.
